# Cell-Free Nucleic Acids

**DOI:** 10.3390/ijms20225645

**Published:** 2019-11-12

**Authors:** Balint Nagy

**Affiliations:** Department of Human Genetics, Faculty of Medicine, University of Debrecen, H-4032 Debrecen, Hungary; nagy.balint@med.unideb.hu

The discovery of cell-free DNA (cfDNA) dates back to 1948, when Mandel and Metais found it in the sera of cancer patients [1]. Later, Tan et al. observed a correlation with the cfDNA concentration and development of autoimmune disease in 1966 [2]. Leon et al. started to use cfDNA in tumor diagnosis in 1977, but unfortunately due to the molecular biological technical possibilities they were not very successful [3]. A break through occurred in 1997, when Dennis Lo started to detect RhD and fetal sex in maternal plasma by using real-time PCR [4]. The real spread of non-invasive detection of fetal genetic diseases started in 2011, when massive parallel sequencing was introduced [5]. Nowadays, about half of the prenatal genetic examination is performed by so-called non-invasive prenatal testing (NIPT).

The clinical application of cfDNA has been rapidly growing in the field of oncology; it gives the possibility of the early detection of cancer in different body fluids via liquid biopsy. Cancer type specific molecular signatures could be detected in very early stages of tumor development. Drug resistance is a burning problem during the treatment of patients. In addition to the mutation screening methylation profile, there has been increasing interest in the use of surrogate markers for follow up in cancer patients with metastasis [6].

Alongside the increasing application of cfDNA, interest is growing in the utilization of cell-free RNAs (cfRNAs), such as microRNA (miRNA), long non-coding RNA (lncRNA), and circular RNA (circRNA), in different types of diseases [7]. Their concentrations are surprisingly stable in sera or plasma due to their encapsulation into extracellular vesicles (microvesicles, exosomes). From these, it seems that exosomes could tremendously improve the current diagnostic arsenal. While the exact nucleic acid, protein, and lipid contents of these small microvesicles are still under investigation, it has been shown that exosomes play important roles in intracellular, cell-cell, and cell-tissue communication [8].

MiRNAs are important small non-coding RNAs that are 18–25 base pairs (bp) in size. They are able to bind to proteins such as Argonaute-2, HDL, and LDL, and a single miRNA can regulate the expression of several of genes [9,10]. Disturbances in the regulation of key miRNAs can have tremendous effects on gene expression and on normal and pathophysiological processes [6].

LncRNAs, which are >200 bp in size, are new players in this field. It seems that they have an even higher diagnostic and prognostic value due to their specific expression in different type of tissues and diseases; importantly, they are very stable in different conditions [11]. It has been shown that they are useful in the diagnosis of different types of cancer and cardiovascular diseases [11].

CircRNAs are newly discovered non-coding RNAs with the size of couple of thousands base pairs. They have a closed circular structure with a function of tumor suppressor or promoter in several types of cancer. They can be used as biomarkers or therapeutic targets [12]. They can also serve as sponges to inhibit miRNAs [13]. Altered expression of circRNAs has been reported recently [14,15].

This exciting field of research is expected to produce a lot of diagnostic and new generation treatment possibilities. The high interest in this topic shows in the enormous quantity of published papers in different journals. Coincidentally, there was a Special Issue in the *Journal of Biotechnology* earlier this year dealing with this subject. Researchers from Central-Eastern Europe showed their work on cell-free nucleic acids [16,17,18,19].

This Special Issue contains eight original research studies [20,21,22,23,24,25,26,27], and five review papers [28,29,30,31,32]; the diversity of the papers demonstrates how many different topics are covered by cell-free nucleic acid research.

Three research papers deal with the prenatal application of cfDNA. Pös et al.’s “Identification of Structural Variation from NGS-Based Non-Invasive Prenatal Testing” shows that copy number variants (CNVs) are important subjects for the study of human genome variations, as CNVs can contribute to population diversity and human genetic diseases [20]. Additionally, CNVs are useful in NIPT, as they are a source of population specific data [20].

Gazdarica et al. studied the reliability of NIPT, which depends on the accurate estimation of fetal fraction [21]. They propose several improvements in fetal fraction estimation to get more reliable results [21].

In their other work, Gazdarica et al. demonstrated a new, more stable prediction method for NIPT that provides highly divergent inter-sample coverage [22].

Preeclampsia is a mysterious disease—despite intensive research, we still do not know the exact details of its development. It seems that cell-free nucleic acids could serve as biomarkers for the early detection of this disease. Hromadnikova et al. measured exosomal C19MC microRNAs and found them to be important 6in the detection of pregnancy associated complications [23]. 

Improving the success rate of vitro fertilization (IFV) and embryo transfer (ET) is an important goal. Timofeeva et al. reported a very interesting application of small non-coding RNAs to improve the efficiency of embryo transfer (ET) by measuring embryo-specific sncRNAs in the culture media [24].

Ovarian cancer is one of the leading serious malignancies among women, with high incidence of mortality; the introduction of new diagnostic markers could help in its early detection and treatment. Penyige et al. showed their results from using the NanoString technique to get information on the expression of 800 miRNAs in one run, and they checked the reliability of the obtained results by conventional real-time PCR [25].

Epigenetic regulation is very important during the development of diseases and drug resistance. Dvorská et al. found that methylation changes are important signs during ovarian cancer development and that the CDH1 gene is a potential candidate for being a non-invasive biomarker in the diagnosis of ovarian cancer [26].

We received interesting reviews on the application of cell-free nucleic acids. Zubor at al. reviewed the deficits of mammography and demonstrated the potential of non-invasive diagnostic testing using circulating miRNA profiles [27].

Exosomes are important in the transfer of genetic information. Konečná et al. discussed the current knowledge on not only exosome-associated DNA but on vesicles-associated DNA, and their role in pregnancy-related complications [28]. It seems that a major obstacle is the lack of a standardized technique for exosomes isolation and measurement [28].

Kubiritova et al. summarized what we know about cell-free nucleic acids in inflammatory bowel disease (IBD). Despite extensive research, the etiology and exact pathogenesis are still unclear, although similar to the cfNAs (cell-free ribonucleic acids) observed in other autoimmune diseases, it seems to be relevant in IBD. The authors collected literature on cfDNA and cfRNA and on exosomes and neutrophil extracellular traps and their association with IBD. Based on the information from the reported literature, they propose the use of cfNAs (cell-free nucleic acids) in the management of IBD as biomarkers and as a potential therapeutic target [29].

Dvorska et al. reviewed the utility of liquid biopsy as a tool for the differentiation of leiomyomas and sarcomas of corpus uteri [30]. They collected the most important knowledge of mesenchymal uterine tumors and showed the benefits of liquid biopsy [30].

Microchimerism has also recently become a hot topic too. Andrikovics et al. discuss microchimerism in the context of various forms of transplantation and transplantation-related advanced therapies, and they show the available cfNA (cell-free nucleic acid) markers and detection platforms [31].

There is only one article in this issue related to animal studies. Janovičová et al. showed that sex, age, and bodyweight are not determinants of cfDNA variability in healthy mice, and they call attention to the importance of understanding the production and cleavage of cfDNA [32].

I would like express my thanks to all of the authors for their valuable contributions to this Special Issue, and would also like to express my gratitude to the editorial staff members and anonymous reviewers who helped to improve the quality of the submitted manuscripts. I hope readers will find this issue to be both interesting and useful.
